# Vessel‐associated microglia are differentially activated and distributed in relation to systemic infection and Alzheimer's disease

**DOI:** 10.1111/bpa.70052

**Published:** 2025-11-30

**Authors:** Oliver Milner, Robert A. Fisher, Daniel J. Asby, Stephen Cross, Delphine Boche, J. Scott Miners

**Affiliations:** ^1^ Cerebrovascular and Dementia Research Group, Translational Health Sciences, Bristol Medical School University of Bristol Bristol UK; ^2^ Wolfson Bioimaging Facility, Faculty of Health and Life Sciences University of Bristol Bristol UK; ^3^ Clinical Neurosciences, Clinical and Experimental Sciences, Faculty of Medicine University of Southampton Southampton UK

**Keywords:** blood–brain barrier, microglia, neuroinflammation, neurovascular, systemic infection, vascular‐associated microglia

## Abstract

Vessel‐associated microglia (VAM) are an integral part of the neurovascular unit and have recently been implicated in the pathophysiology of cerebrovascular injury and blood–brain barrier (BBB) leakiness in Alzheimer's disease (AD). In this neuropathological study, we explored the hypothesis that the distribution and activation of VAM are altered in AD in the presence of systemic infection, associated with cerebrovascular dysfunction. We studied VAM density in the temporal cortex and underlying white matter from AD and age‐matched controls with and without terminal systemic infection (SI) (*n* = 15 per group). The area of VAM labelled with microglial markers (Iba1, HLA‐DR, CD68) was quantified in proximity to CD31‐labelled microvessels within three predefined regions: contact VAM, proximity <15 μm, and parenchymal >15 μm. The relationships between VAM and previously measured brain cytokine levels and biochemical markers of cerebral perfusion (MAG:PLP1, endothelin‐1) and BBB leakiness (VEGF‐A and fibrinogen), were explored in a subset of cases. Compared to controls, the relative area of Iba1+ VAM was higher in SI and in AD. The area of HLA‐DR+ VAM was higher in AD only. The area of Iba1+ VAM that expressed CD68, a marker of phagocytosis, was higher in both AD and AD + SI. Iba1+ and HLA‐DR+ VAM correlated inversely with anti‐inflammatory cytokines (IL‐10, IL‐23) in AD and positively with pro‐inflammatory cytokines (IL‐6, IL‐23, GM‐CSF, IL‐17) in AD + SI. Iba1+ VAM density correlated positively with endothelin‐1, VEGF‐A and fibrinogen in controls. HLA‐DR+ VAM density correlated positively with Aβ_1‐42_ in both controls and AD, and inversely with PDGFRβ and VCAM‐1 in AD. Our data reveal the distribution of VAM is elevated in AD, and altered in the presence of systemic infection, which together are likely to be independent and synergistic contributors to cerebrovascular dysfunction in AD.

## Graphical abstract

This post‐mortem study revealed an increased density of vessel‐associated microglia (VAM) in the temporal lobe in systemic infection and Alzheimer's disease. VAM density was related to brain cytokine levels and markers of cerebrovascular dysfunction and BBB leakage indicating that activated VAM contributes to neurovascular injury in Alzheimer's disease.

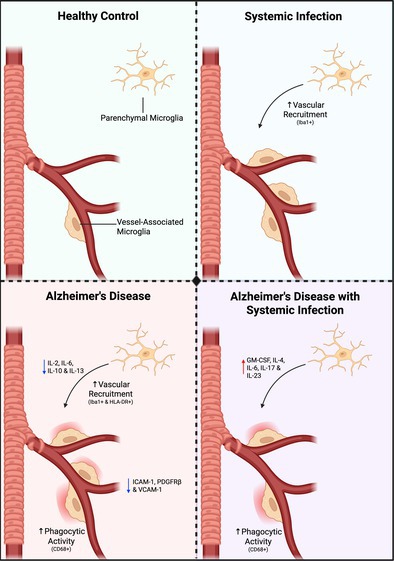

## 1 INTRODUCTION

Alzheimer's disease (AD) is characterized by amyloid‐beta (Aβ) plaque and neurofibrillary tangle pathology but often co‐exists with cerebrovascular pathology including cerebral amyloid angiopathy and cerebral small vessel disease. Cerebrovascular dysfunction, such as reduced cerebral blood flow [1] and blood–brain barrier (BBB) breakdown [2], are early pathogenic features of AD linked to cognitive decline; and Aβ and tau pathology [3]. We have previously shown that systemic infection, related to neuroinflammation, exacerbates cerebrovascular dysfunction and injury independently of Aβ in AD [4]. In this study, we explored the hypothesis that cerebrovascular dysfunction associated with systemic infection is mediated by the activation and clustering of vascular‐associated microglia (VAM) in proximity to cerebral blood vessels.

The neurovascular unit is comprised of endothelial cells, mural cells (either pericytes or smooth muscle cells), astrocytic end‐feet, and neurons that regulate cerebral blood flow, BBB integrity, and immune cell entry to the brain. In the adult mouse brain, up to 30% of microglia are vessel‐associated [5] and surround the outside of the blood vessels, interspersed between astrocytic end‐feet [6]. The nomenclature of these cells is varied and refers to capillary‐associated microglia [5], juxtavascular [7], pericyte‐associated [8], perivascular [9] or vessel‐associated microglia (VAM) [10]. In the present study, we refer to these cells as VAM. VAM regulate cerebral blood flow [5] and BBB permeability [10] and migrate along the capillary wall to restore homeostatic function compensating for astrocytic end feet retraction [11, 12]. In response to capillary injury, VAM can extend their processes around injured cerebral blood vessels and mediate vascular repair restoring BBB integrity in mouse models of cerebrovascular injury [13, 14]. Pharmacological ablation of microglia, or inhibition of the P2RY12 receptor, impairs neurovascular coupling (reviewed [15]). Together, these studies highlight an essential role of VAM as modulators of neurovascular function.

Recent experimental studies have shown that under chronic inflammatory conditions, VAM are implicated in cerebrovascular breakdown by disrupting neurovascular coupling. During sustained systemic inflammation, VAM adopt a phagocytic phenotype engulfing astrocytic end feet resulting in BBB leakiness [10]. VAM also induce acute endothelial necroptosis [16], stimulate pericytes to produce MMP‐9 (associated with BBB breakdown) [17] and polarise astrocytes to adopt a proinflammatory phenotype [18]. VAM retain a phagocytic profile long after the reversal of hypoxic conditions in 20‐month‐old mice exposed to chronic low‐level oxygen [19]. Together, these studies reveal that VAM contribute to cerebrovascular pathology in conditions associated with chronic low‐level infection and inflammation. Mediators released from injured endothelial cells induce the migration of CNS microglia, and circulatory immune cells, towards the cerebrovasculature in response to systemic infection [10, 20].

Systemic infection (SI) promotes neuroinflammation and exacerbates cognitive decline associated with small vessel disease and AD. Elevated levels of serum IL‐1β [21] are linked to brain tissue injury via activation of microglia, vascular macrophages [22, 23] and endothelial cell injury [24]. Elevated CD68‐labelled microglia, dysregulation of brain cytokine levels, and T‐cell recruitment, have all been shown in AD with systemic infection independently of parenchymal Aβ or Tau [25]. Our previous study revealed that markers of cerebrovascular injury, including brain tissue perfusion and BBB leakiness, were exacerbated in AD with SI, associated with elevated levels of pro‐inflammatory cytokines within the brain, independently of Aβ pathology [4]. In the present study, we have explored the relationship between systemic infection and cerebrovascular injury in AD, by mapping the distribution and phenotype of VAM across four experimental groups: Controls +/− SI and AD +/− SI. We have also explored whether the distribution of VAM is altered in relation to brain cytokine levels and markers of cerebral hypoperfusion and BBB leakiness, measured in a subset of cases from a previous study [4].

## 2 METHODS

### 2.1 Cohort

Human formalin‐fixed, paraffin‐embedded (FFPE) brain tissue from the superior temporal cortex (BA22) and underlying white matter was provided under the ethical approval of the South West Dementia Brain Bank (SWDBB), University of Bristol. AD and age‐matched controls were stratified according to the presence or absence of terminal systemic infection (SI) (+/− SI, *n* = 15 per group). AD cases had a clinical diagnosis of AD during life with intermediate or high AD neuropathological change according to the NIA‐AA guidelines [26]. Control brains were obtained from individuals without a clinical history of dementia and characterized by few or absent neuritic plaques and a Braak tangle stage of III or less with no other neuropathological abnormalities. A summary of the demographics of the cohort is shown in Table 1. A list of the UK brain bank identifier numbers for individual cases is provided in Table S1. Information on the death certificate was used to identify cases with terminal systemic infection recorded as the primary cause of death. The types of systemic infection listed as the primary cause of death for cases in this study are shown in detail in Table S2, and overlap with cases previously reported [4].

**TABLE 1:** Study cohort.

	Con	Con + SI	AD	AD + SI
*N*	15	15	15	15
Age (*M* ± SD)	86.2 (±6.2)	86.2 (±7.4)	82.0 (±4.9)	82.2 (±5.3)
PM delay hours (*M* ± SD)	40.1 (±20.7)	46.7 (±13.5)	39.4 (±20.3)	40.8 (±17.0)
Braak tangle stage	0–III	0–III	IV–VI	III–VI
Female	8	7	8	9
Male	7	8	7	6

*Note*: Cohort demographics. Summary table of the demographics of the tissue cohort.

Abbreviations: AD, Alzheimer's disease; AD + SI, Alzheimer's disease with terminal systemic infection; Con, control; Con + SI, control with terminal systemic infection; *M*, mean; *N*, number of cases; SD, standard deviation.

### 2.2 Immunofluorescence labelling of vessel‐associated microglia

To dewax, FFPE tissue sections (7 μm) were placed in a 60°C oven for a minimum of 1 h before submerging in clearene twice for 5 min, then twice in 100% ethanol for 5 min. Tissue sections were rehydrated in running water. For antigen retrieval, the sections were microwaved at high power for 10 min in Tris‐EDTA buffer, pH 9. Sections were then blocked for 30 min in 5% donkey serum in PBS at room temperature (RT) before adding primary antibodies, which were incubated overnight at 4°C at the following dilutions of stock concentration (0.2 mg/mL): CD31 (1:150, R&D Systems, AF806); CD68 (1:75, Dako, M0876); HLA‐DR (1:500, Abcam, AB20181); and IBA1 (1:1000, AlphaLabs Wako, 019‐19741). The corresponding secondary antibodies were incubated for 1 h at RT using the following dilutions: donkey anti‐mouse:555 (1:500, Invitrogen, A31570); donkey anti‐rabbit:488 (1:500, Invitrogen, A21206); and donkey anti‐sheep:405 (1:350, Abcam, AB175676). To reduce tissue autofluorescence, Autofluorescence Eliminator Reagent (Millipore, 2160) and TrueView Autofluorescence Quenching Kit (Vector, SP‐8500‐15) were sequentially applied to the tissue for 5 min each.

### 2.3 Confocal microscopy

Images were captured at ×20 magnification using an Olympus FV3000 confocal microscope. For each case, eight images were taken: four images from randomly selected areas of grey matter and four images from randomly selected areas of white matter within the same section, with six‐times line averaging at a resolution of 2048 × 2048 px. Representative images of CD31, CD68 and Iba1 and CD31, HL‐ADR, and Iba1 within the pre‐defined regions of temporal cortex and underlying cortex are shown in Figures S1 and S2, respectively.

### 2.4 Image analysis

An ImageJ plugin was developed in‐house to quantify the area related to immunofluorescence‐labelled microglia within predefined concentric zones (zone < 1 μm, 1 μm < zone < 15 μm, zone > 15 μm) around CD31+ microvessels applying a minimum size exclusion threshold of 10 μm^2^ to selectively label the microvasculature (see Figure S3 for installation instructions) [27, 28]. To determine the relative area of VAM, the total pixel area of target protein expression within these zones was normalised to the total pixel area of the zone it was detected within. Microglia (Iba1, HLA‐DR, CD68) were defined as: (i) within <1 μm or overlapping with the CD31+ cells to be ‘contacting’ VAM; (ii) between 1 and 15 μm from microvessels, based on criteria previously published [5] as ‘proximity’ VAM; and (iii) > 15 μm from vessels as parenchymal microglia. Unless otherwise stated, we refer to ‘contact’ VAM more generally as VAM throughout the study.

### 2.5 Biochemical assessment of cytokine levels and cerebrovascular markers

Brain cytokine levels were previously measured in brain tissue homogenates using the V‐Plex MSD Proinflammatory Human Protein Panel (cat. no. K15049D) and Cytokine Human Protein Panel (cat. no. K15050D), on the V‐Plex MSD electrochemiluminescence multi‐spot assay platform (MesoScale Diagnostics) and have been detailed in a previous study [4]. Fibrinogen levels in brain tissue homogenates, used as a proxy marker of BBB leakiness; MAG:PLP1 ratio, an antemortem marker of oxygenation of post‐mortem brain tissue; VEGF‐A, as an acute marker of brain ischaemia; endothelin‐1 (EDN1), a potent vasoconstrictor, and CD31 and PDGFRB to assess endothelial and pericyte content, respectively, were also previously measured by ELISA in overlapping cases used in this study [4].

### 2.6 Statistical analysis

To test for statistical significance between total pixel area of microglia markers per concentric zone, adjusted for area, in AD and/or the presence of terminal systemic infection, we used two‐way ANOVA. We used Pearson's correlation coefficient test to assess linear correlations between VAM labelling density and biochemical markers of cytokines and cerebrovascular function. Statistical analysis was performed using GraphPad Prism version 10.2.0 (GraphPad Software, La Jolla, CA) and SPSS version 29.0.2 (SPSS, Chicago). *p*‐values <0.05 were considered statistically significant. Data points exceeding ±2 standard deviations from the mean were considered outliers and excluded from further analysis. The number of outliers removed is shown within the figure legends for each individual analysis.

## 3 RESULTS

### 3.1 Iba1+ VAM density is elevated in Alzheimer's disease and higher in controls with systemic infection

**FIGURE 1:**
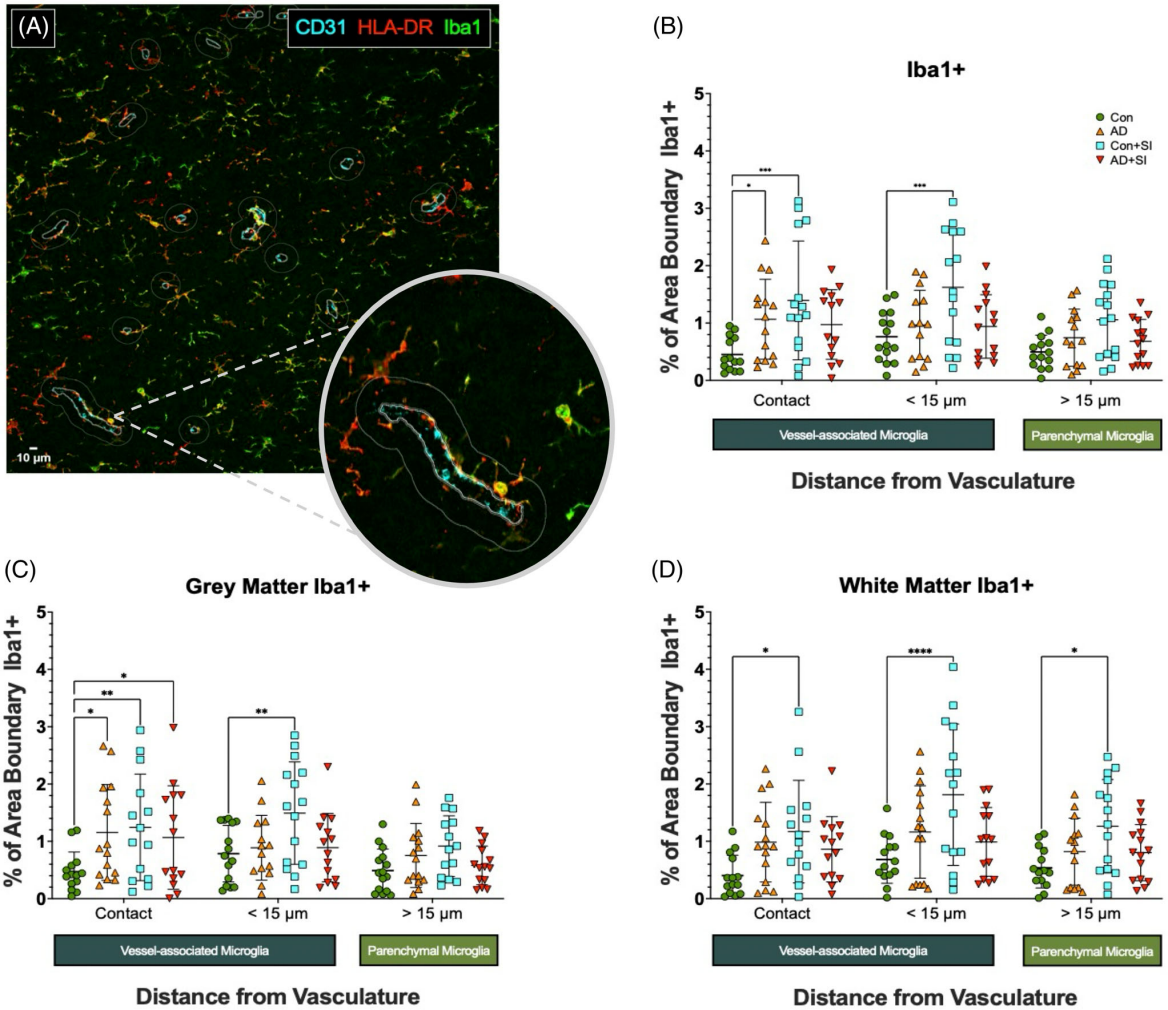
Iba1+ vessel‐associated microglia (VAM) density is increased in systemic infection and Alzheimer's disease. (A) Representative ×20 magnification image of CD31 (cyan), HLA‐DR (red) and Iba1 (green) immunofluorescent labelled sections of temporal lobe with optical zoom overlay showing VAM contact with the vasculature and the three user‐defined regions. (B) Dot plot showing the overall area of Iba1+ labelling within pre‐defined boundaries (contact VAM <1 μm; proximity VAM < 15 μm; parenchymal microglia >15 μm) in Con, AD, Con + SI, and AD+SI (*n* = 15 per group). Outliers removed: contact (C = 1, AD = 0, C + SI = 0, AD + SI = 1), <15 μm (C = 0, AD = 1, C + SI = 0, AD + SI = 1), >15 μm (C = 1, AD = 1, C + SI = 0, AD+SI = 1). (C) Dot plot showing Iba1+ labelling within pre‐defined regions within the temporal lobe grey matter. Outliers removed: contact (C = 1, AD = 0, C + SI = 1, AD + SI = 1), <15 μm (C = 0, AD = 1, C + SI = 0, AD + SI = 1), >15 μm (C = 0, AD = 0, C + SI = 1, AD + SI = 1). (D) Dot plot showing Iba1+ labelling within pre‐defined regions within the underlying white matter. Outliers removed: contact (C = 1, AD = 0, C + SI = 1, AD + SI = 1), <15 μm (C = 1, AD = 0, C + SI = 0, AD + SI = 1), >15 μm (C = 1, AD = 1, C + SI = 0, AD + SI = 0). AD, Alzheimer's disease; AD + SI, Alzheimer's disease with terminal systemic infection; CD31, cluster of differentiation 31; Con, control; Con + SI, control with terminal systemic infection; Iba1+, ionised calcium‐binding adaptor molecule 1 positive; VAM, vessel‐associated microglia. *p < 0.05. **p < 0.01. ***p < 0.001. ****p < 0.0001.

Representative images of Iba1+ microglia in proximity with CD31 labelled vessels are shown in Figure 1A. Compared to non‐disease controls (Con), the total density of Iba1+ ‘contacting’ VAM was higher in AD (*p* < 0.05) and Con + SI (*p* < 0.001) compared to Con only (Figure 1B). No difference was observed in AD with and without systemic infection (SI). Iba1+ VAM density was higher in Con + SI compared to Con only (*p* < 0.001) in the <15 μm ‘proximity’ region. No significant difference was observed for Iba1+ microglia within the ‘parenchymal’ >15 μm zone between groups.

When analysed by region, the density of contacting Iba1+ VAM was higher in AD (*p* < 0.05), AD + SI (*p* < 0.05), and Con + SI (*p* < 0.01) compared to Con within the cortex (Figure 1C). The density of Iba1+ VAM within the <15 μm ‘proximity’ region was higher in Con + SI than Con (*p* < 0.01). Iba1+ VAM density was unaltered in the >15 μm ‘parenchyma’ region.

In the underlying white matter, Iba1+ density was unchanged in AD and AD+SI compared to controls across all regions. Iba1+ density was, however, significantly higher in Con + SI compared to Con in the contact (*p* < 0.05), proximity (*p* > 0.0001) and parenchymal (*p* < 0.05) regions (Figure 1D).

### 3.2 HLA‐DR+ VAM density is increased in Alzheimer's disease but not systemic infection

**FIGURE 2:**
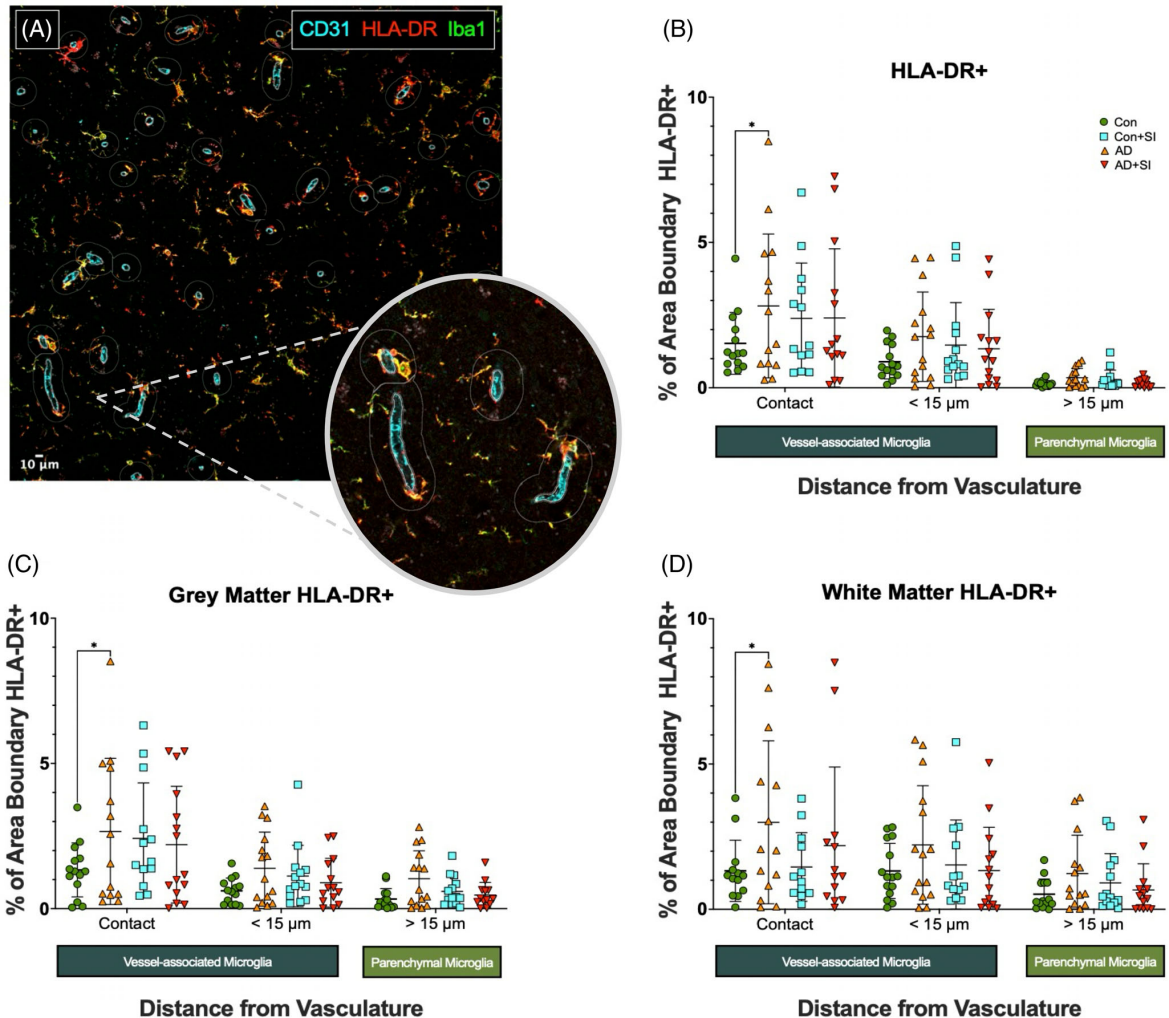
HLA‐DR+ VAM are increased in Alzheimer's disease. (A) Representative ×20 magnification image of CD31 (cyan), HLA‐DR (red) and Iba1 (green) immunofluorescent labelled sections of temporal lobe with optical zoom overlay showing VAM contact with the vasculature. (B) Dot plot showing the overall area of HLA‐DR+ density within pre‐defined regions in Con, AD, Con + SI and AD + SI (*n* = 15 per group). Outliers removed: contact (C = 1, AD = 1, C + SI = 2, AD + SI = 1), <15 μm (C = 1, AD = 0, C + SI = 1, AD + SI = 0), >15 μm (C = 1, AD = 1, C + SI = 1, AD + SI = 2). (C) Dot plot showing HLA‐DR+ labelling within pre‐defined regions within the temporal lobe grey matter. Outliers removed: contact (C = 1, AD = 1, C + SI = 2, AD + SI = 0), <15 μm (C = 1, AD = 0, C + SI = 1, AD + SI = 1), >15 μm (C = 1, AD = 0, C + SI = 1, AD + SI = 2). (D) Dot plot showing HLA‐DR+ labelling within pre‐defined regions within the underlying white matter. AD, Alzheimer's disease; AD + SI, Alzheimer's disease with the presence of systemic infection. Outliers removed: contact (C = 2, AD = 1, C + SI = 3, AD + SI = 2), <15 μm (C = 0, AD = 0, C + SI = 2, AD + SI = 1), >15 μm (C = 1, AD = 1, C + SI = 1, AD + SI = 1); CD31, cluster of differentiation 31; Con, control; Con + SI, control with the presence of systemic infection; HLA‐DR+, human leukocyte antigen‐DR positive; VAM, vessel‐associated microglia. *p < 0.05.

To investigate whether the distribution of ‘active’ antigen‐presenting VAM differed in AD and were altered by the presence of systemic infection, we assessed contacting HLA‐DR+ VAM density in proximity to CD31 labelled blood vessels, as shown by representative images (Figure 2A). Overall, the density of contacting HLA‐DR+ VAM was higher in AD compared to Con in temporal cortex and white matter combined (*p* < 0.05) (Figure 2B), and in the cortex and underlying white matter separately (*p* < 0.05 for both) (Figure 2C,D). HLA‐DR+ contacting VAM was unchanged between AD and AD + SI and was not significantly elevated in Con + SI compared to Con alone. HLA‐DR+ VAM density in the <15 μm ‘proximity’ region or within the >15 μm ‘parenchymal’ region did not differ between groups in either cortex or white matter.

### 3.3 Phagocytic marker CD68 is elevated in VAM in Alzheimer's disease

**FIGURE 3:**
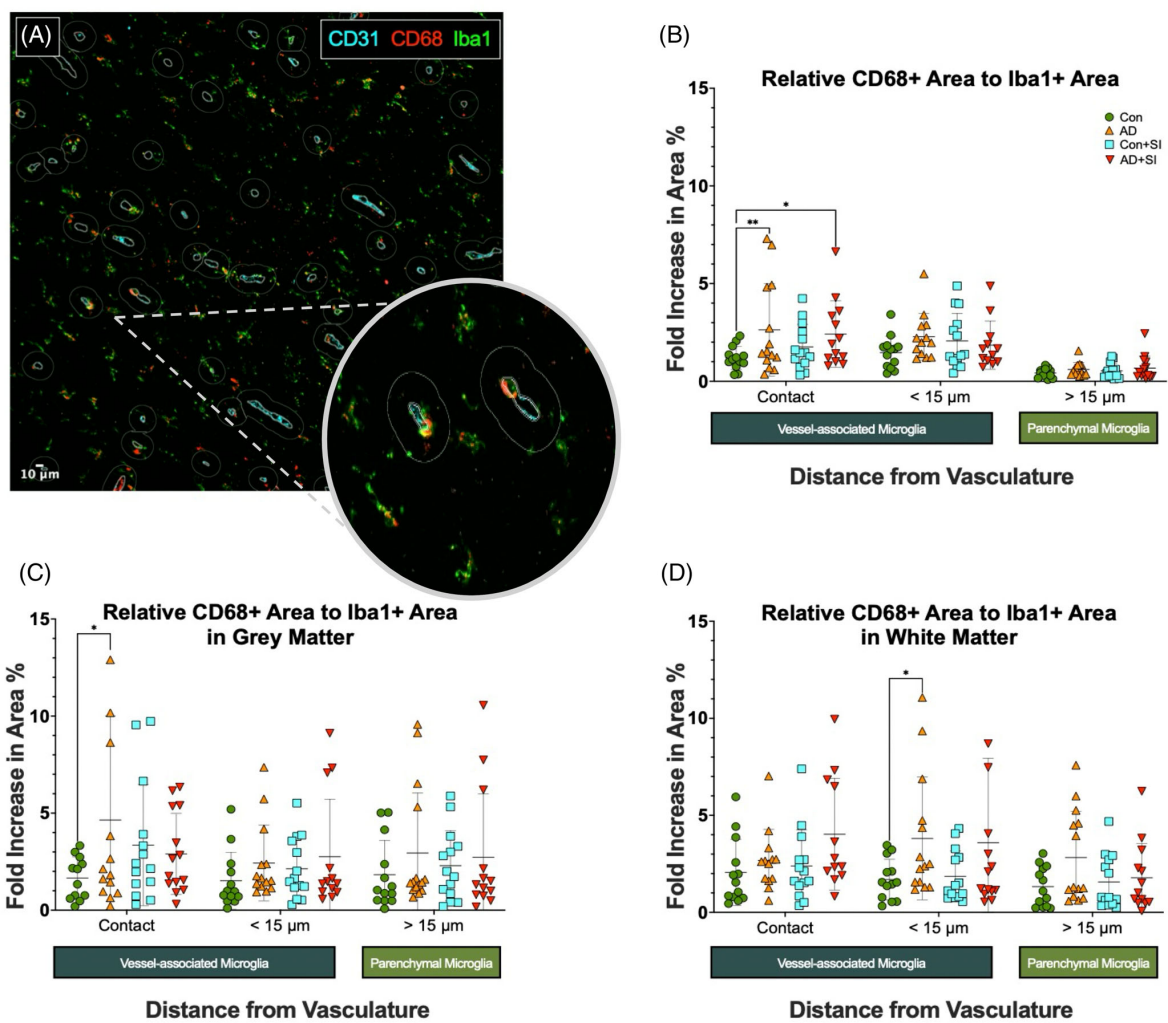
VAM labelled CD68 is elevated in Alzheimer's disease. (A) Representative ×20 magnification image of CD31 (cyan), CD68 (red) and Iba1 (green) immunofluorescent labelled sections of temporal lobe with optical zoom overlay showing CD68+ expression within Iba1+ microglia around the vasculature. (B) Dot plot showing the relative change in CD68+ area compared to Iba1+ area within pre‐defined regions of temporal cortex in Con, AD, Con + SI, AD + SI (*n* = 15 per group). Outliers removed: contact (C = 2, AD = 1, C + SI = 1, AD + SI = 2), <15 μm (C = 2, AD = 22, C + SI = 1, AD + SI = 3), >15 μm (C = 2, AD = 2, C + SI = 1, AD + SI = 1). (C) Dot plot showing the relative change in CD68+ area compared to Iba1+ area within pre‐defined regions within the temporal lobe grey matter. Outliers removed: contact (C = 3, AD = 1, C + SI = 1, AD + SI = 1), <15 μm (C = 2, AD = 1, C + SI = 1, AD + SI = 2), >15 μm (C = 2, AD = 0, C + SI = 1, AD + SI = 2). (D) Dot plot showing the relative change in CD68+ area compared to Iba1+ area within pre‐defined regions within the underlying white matter. Outliers removed: contact (C = 2, AD = 2, C + SI = 1, AD + SI = 3), <15 μm (C = 2, AD = 1, C + SI = 1, AD + SI = 1), >15 μm (C = 2, AD = 1, C + SI = 1, AD + SI = 2). AD, Alzheimer's disease; AD + SI, Alzheimer's disease with the presence of systemic infection; CD31, cluster of differentiation 31; CD68, cluster of differentiation 68; Con, control; Con + SI, control with the presence of systemic infection; Iba1+, ionised calcium‐binding adaptor molecule 1 positive; VAM, vessel‐associated microglia. *p < 0.05. **p < 0.01.

To determine if the phagocytic phenotype of VAM was altered in AD and was related to the presence of systemic infection, we co‐labelled Iba1 with CD68, and calculated the CD68 positive area relative to the total Iba1+ labelled area within each of the three zones (Figure 3A). The CD68+ area was significantly higher in AD (*p* < 0.01) and AD + SI (*p* < 0.05) compared to Con within the contact zone (Figure 3B) but did not differ between Con and Con + SI. CD68+ VAM area also did not differ between groups in the <15 and >15 μm regions.

In the cortex, the CD68+ adjusted area within the contact zone was increased in AD only (*p* < 0.05) (Figure 3C). In white matter, it was higher in AD compared to Con (*p* < 0.05) in the <15 μm region only (Figure 3D). The levels of unadjusted CD68 raw data are shown in Figure S4.

The relative changes in Iba1+, HLA‐DR+, and CD68‐labelled microglia within the pre‐defined contact, proximity and parenchymal zones in AD, AD + SI and Con + SI compared to Con, are summarised in Table 2.

**TABLE 2:** Overview of altered VAM density in the presence of systemic infection and Alzheimer's disease.

	Iba1+			HLA‐DR+			Relative CD68 to Iba1+ area		
	Contact	<15 μm	>15 μm	Contact	<15 μm	>15 μm	Contact	<15 μm	>15 μm
Con + SI									
Combined	***	***							
Grey matter	**	**							
White matter	*	****	*						
AD									
Combined	*			*			**		
Grey matter	*			*			*		
White matter				*				*	
AD + SI									
Combined							*		
Grey matter	*								
White matter									

*Note*: Summary of VAM‐labelling in systemic infection and Alzheimer's disease compared to controls. Statistically significant differences between the density of labelled VAM when compared to controls are shown for each of the pre‐defined regions. In all cases, the differences represent an increase in density compared to controls. *p < 0.05; **p < 0.01; ***p < 0.001; ****p < 0.0001.

Abbreviations: AD, Alzheimer's disease; AD + SI, Alzheimer's disease with terminal systemic infection; CD68/, cluster of differentiation 68; Con + SI, control with terminal systemic infection; HLA‐DR+, human leukocyte antigen‐DR positive; Iba1, ionised calcium‐binding adaptor molecule 1; Iba1+, ionised calcium‐binding adaptor molecule 1 positive; VAM, vessel‐associated microglia.

### 3.4 Contacting VAM density is differentially related to brain cytokine levels in AD and AD with systemic infection

We next assessed the correlations between the area of Iba1+ and HLA‐DR+ VAM with previously measured levels of brain cytokines in brain tissue homogenates from a subset of cases that overlapped with our previous study [4].

**FIGURE 4:**
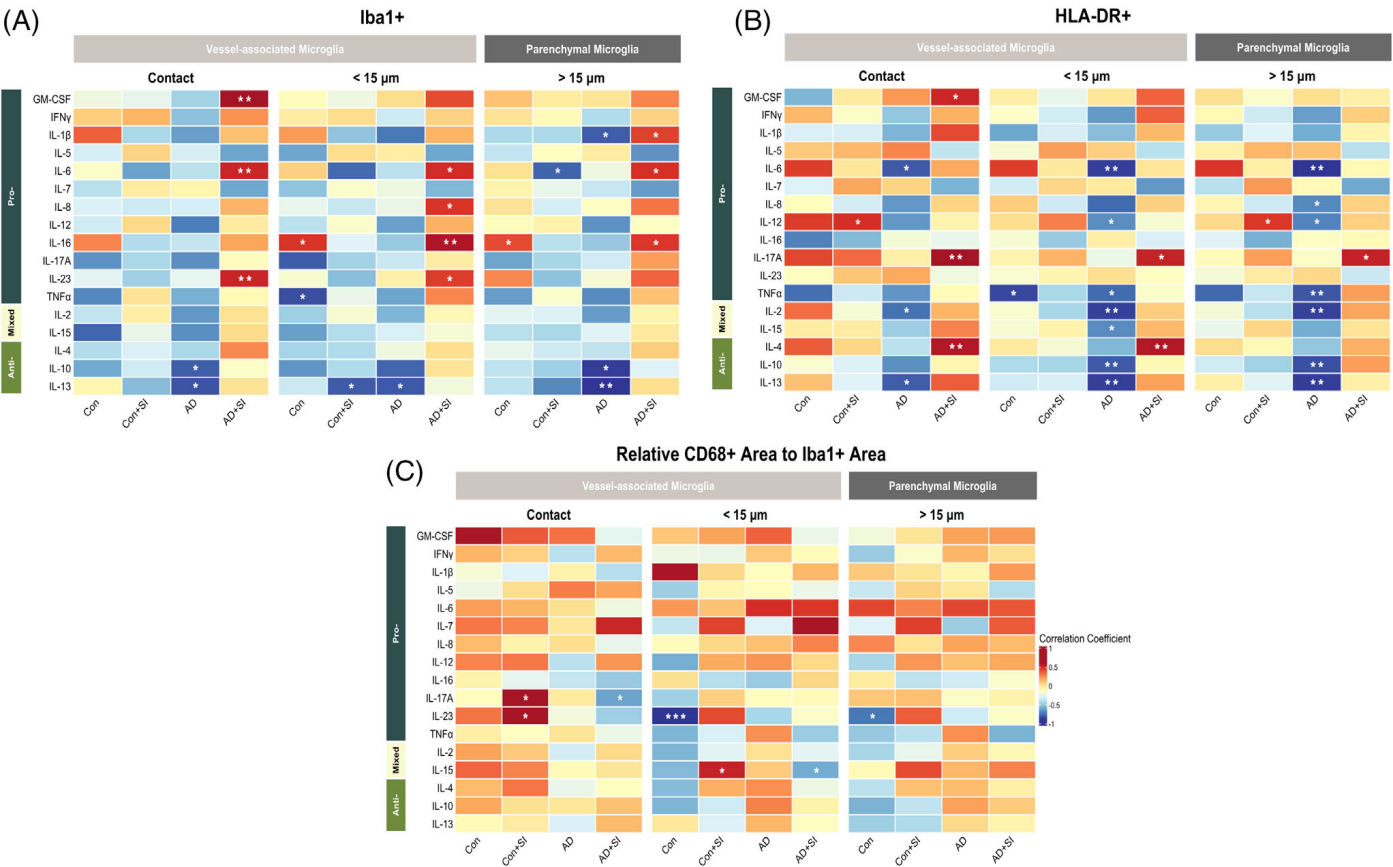
Heatmaps showing the relationships between VAM and brain cytokine levels. Heatmaps illustrate the relationship between (A) Iba1+ (B) HLA‐DR+ and (C) CD68+ adjusted Iba1+ area in relation to pro‐ and anti‐inflammatory brain cytokine levels within each pre‐defined region. Cytokines are organised according to their primary role but many have both pro‐ and anti‐inflammatory actions that are context‐dependent. AD, Alzheimer's disease; AD + SI, Alzheimer's disease with the presence of systemic infection; CD68, cluster of differentiation 68; Con, control; Con + SI, control with the presence of systemic infection; GM‐CSF, granulocyte‐macrophage colony‐stimulating factor; HLA‐DR+, human leukocyte antigen‐DR positive; Iba1+, ionised calcium‐binding adaptor molecule 1 positive; IFNγ, interferon gamma; IL‐, interleukin; TNFα, tumour necrosis factor alpha. *p < 0.05. **p < 0.01. ***p < 0.001.

The correlations between Iba1+ VAM area and brain cytokine levels are summarised as a heatmap (Figure 4A). The density of Iba1+ contacting VAM area was unrelated to cytokine levels in Con and Con + SI. In AD, the density of Iba1+ VAM area correlated *inversely* with anti‐inflammatory cytokines: IL‐10 (*r* = −0.58, *p* < 0.05) and IL‐13 (*r* = −0.61, *p* < 0.05). In AD + SI, Iba1+ VAM area correlated *positively* with pro‐inflammatory cytokines, reaching significance for GM‐CSF (*r* = 0.81, *p* < 0.01), IL‐6 (*r* = 0.71, *p* < 0.01) and IL‐23 (*r* = 0.71, *p* < 0.01). Within the parenchyma only, Iba1+ density correlated inversely with IL‐1β (*r* = −0.58, *p* > 0.05) in AD and positively with IL‐1β (*r* = 0.63, *p* > 0.05) in AD + SI.

HLA‐DR+ contacting VAM area correlated positively with IL‐12 level, approaching significance in Con (*r* = 0.60, *p* = 0.06), and reaching significance in Con + SI (*r* = 0.63, *p* < 0.05). HLA‐DR+ VAM correlated *inversely* and weakly with the majority of brain cytokines in AD: IL‐2 (*r* = −0.60, *p* < 0.05), IL‐6 (*r* = −0.66, *p* = 0.05), IL‐8 (*r* = −0.50, *p* = 0.069), IL‐10 (*r* = −0.52, *p* = 0.057), IL‐12 (*r* = −0.50, *p* = 0.067), IL‐13 (*r* = −0.65 *p* < 0.05), and TNF‐α (*r* = −0.50, *p* = 0.065). The strength of these inverse correlations was greater in both <15 and >15 μm regions in AD (Figure 4B). In contrast, HLA‐DR+ VAM correlated *positively* with GM‐CSF (*r* = 0.66, *p* < 0.05), IL‐4 (*r* = 0.73, *p* < 0.01) and IL‐17A (*r* = 0.77, *p* < 0.01) in the AD + SI group. These relationships were strongest within the contact area and lower or absent in <15 and >15 μm regions.

The relative area of CD68+, adjusted for Iba1+ area, did not correlate with most of the brain cytokines with the exception of IL‐17A (*r* = 0.63, *p* < 0.05) and IL‐23 (*r* = 0.63, *p* < 0.05) in the Con + SI group, and an inverse correlation with IL‐17A in AD + SI (*r* = −0.62, *p* < 0.05) (Figure 4C).

### 3.5 VAM density is related to markers and mediators of cerebral perfusion, BBB leakiness and Aβ_1‐42_ concentration

Lastly, we explored the relationship between Iba1+ and HLA‐DR+ VAM and biochemical markers of brain tissue oxygenation (MAG:PLP1 ratio), cerebral ischemia (VEGF‐A), vasoconstriction (endothelin‐1; EDN1), BBB leakiness (fibrinogen), pericyte (PDGFRβ) and endothelial (VCAM‐1) dysfunction, measured previously in brain tissue homogenates [4].

**FIGURE 5:**
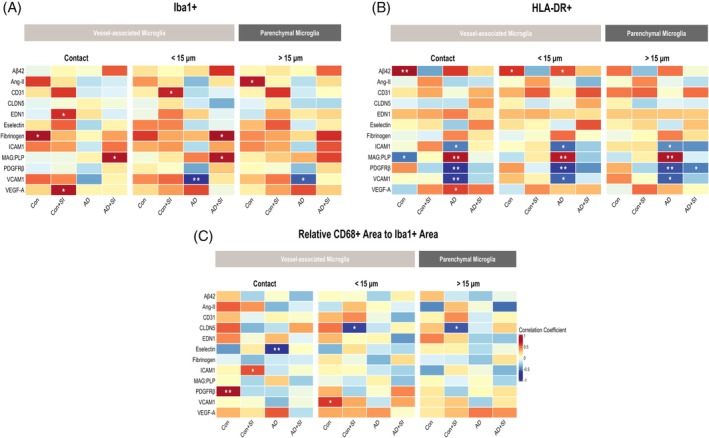
Heatmaps showing the relationships between VAM and biochemical markers of cerebrovascular function. Heatmaps illustrate the relationships between (A) Iba1+ (B) HLA‐DR+ and (C) CD68+ adjusted to Iba1+ area with biochemical markers of cerebrovascular function within the three pre‐defined regions. Aβ42, amyloid beta 42; AD, Alzheimer's disease; AD + SI, Alzheimer's disease with the presence of systemic infection; Ang‐II, angiotensin II; CD31, cluster of differentiation 31; CD68, cluster of differentiation 68; CLDN5, claudin‐5; Con, control; Con + SI, control with the presence of systemic infection; EDN1, endothelin‐1; HLA‐DR+, human leukocyte antigen‐DR positive; Iba1, ionised calcium‐binding adaptor molecule 1 positive; Iba1+, ionised calcium‐binding adaptor molecule 1 positive; ICAM1, intracellular adhesion molecule‐1; MAG:PLP, myelin‐associated glycoprotein:proteolipid protein; PDGFRβ, platelet‐derived growth factor receptor beta; VCAM‐1, vascular cell adhesion protein 1; VEGF‐A, vascular endothelial growth factor‐A. *p < 0.05. **p < 0.01.

Iba1+ contacting VAM area correlated positively with fibrinogen levels (*r* = 0.64, *p* < 0.05), indicative of BBB leakiness, in Con and with endothelin‐1 (*r* = 0.53, *p* < 0.05) and VEGF‐A (*r* = 0.62, *p* < 0.05) levels in Con + SI (Figure 5A). No significant correlations were observed between Iba1+ VAM and vascular markers in AD or AD + SI with the exception of the MAG:PLP ratio (*r* = 0.62, *p* < 0.05) in AD + SI (Figure 5A).

HLA‐DR+ contacting VAM area correlated positively with Aβ42 in Con (*r* = 0.74, *p* < 0.01). HLA‐DR+ contacting VAM area correlated inversely with the MAG:PLP ratio (*r* = −0.58, *p* < 0.05) in Con but positively with MAG:PLP (*r* = 0.71, *p* < 0.01) in AD. HLA‐DR+ contacting VAM density correlated inversely with ICAM1 (*r* = −0.55, *p* < 0.05), VCAM1 (*r* = −0.71, *p* < 0.01), and PDGFRβ (*r* = −0.73, *p* < 0.01). No correlations were found between HLA‐DR+ VAM density and biochemical markers in Con and AD in the presence of systemic infection.

The relative area of CD68+ adjusted for Iba1+, within the contact zone, correlated positively with PDGFRβ level in Con (*r* = 0.76, *p* < 0.01) and with ICAM1 level (*r* = 0.55, *p* < 0.05) in Con + SI (Figure 5C). In contrast, the relative area of CD68+ within the contact zone correlated inversely with E‐selectin (*r* = −0.66, *p* < 0.01) in the AD cohort. No relationships were observed in AD + SI.

Heatmaps showing the relationships between unadjusted CD68+ labelling, brain cytokine levels and biochemical markers of cerebrovascular function are shown in Figure S5.

## 4 DISCUSSION

In the present study, we have mapped the spatial distribution and phenotype of VAM in controls and AD, with and without systemic infection (SI), and investigated their relationship to brain cytokine levels, markers and mediators of cerebrovascular (dys)function, and Aβ pathology. We found increased Iba1+ VAM contact area within AD and controls with SI, whereas HLA‐DR and Iba‐1 adjusted CD68+ labelled contacting VAM area was elevated in AD only and was not altered in controls or AD by SI. The density of Iba1+ contacting VAMs correlated inversely with the levels of anti‐inflammatory cytokines (IL‐10 and IL‐13) in AD and positively with pro‐inflammatory cytokine levels (GM‐CSF, IL‐6, IL‐23) in AD + SI. A similar pattern was observed for HLA‐DR+ VAM in AD and AD + SI. Iba1+ VAM correlated with endothelin‐1, VEGF‐A and fibrinogen in controls but not AD and HLA‐DR+ VAM correlated positively with the concentration of parenchymal Aβ_1‐42_, and inversely with pericyte (PDGFRβ) and endothelial (VCAM‐1, ICAM‐1) injury markers in AD. The present study indicates that the clustering and activation status of VAMs are associated with a net shift towards pro‐inflammation in AD and AD + SI (but may involve different mechanisms). The findings build upon recent experimental studies in mouse models indicating that VAM play an important pathophysiological role within the cerebrovasculature, regulated by the presence of systemic infection in AD.

We first determined whether VAM labelled with a panel of markers (Iba1, HLA‐DR, and CD68), were altered in AD and controls, with and without SI. Iba1+, a marker of microglial motility [29], was elevated most strongly in VAM in controls with systemic infection. Studies have shown that cytokines, such as IL‐6, and chemokine pathways, such as CCL2/CCR2, induce the migration of microglia towards the vasculature [13, 30] and that VAM regulate vascular processes such as angiogenesis, BBB function, and neurovascular coupling in a context‐dependent manner [5, 31, 32]. Iba1+ VAM were also increased in the AD cohort. A recent snRNAseq study revealed a close relationship between endothelial and microglial DEGs in AD cases with mixed vascular pathology, indicating that injured endothelial cells can induce the migration of microglia, via P2YR12 and CX3CR1 signalling, towards the cerebral vasculature [33].

In AD, VAM Iba1+, HLA‐DR+ and CD68+ labelling was not increased in the presence of SI. This may indicate that the combination of AD and SI results in dysfunctional over‐stimulated, exhausted or tolerant [34] microglia that are unable to react appropriately to pathogenic stimuli, reflecting findings from a previous post‐mortem study [25]. The relative increase in CD68+ VAM in AD and AD+SI but not in controls with SI confirms a previous report that CD68 expression is induced in AD, rather than in the presence of infection [25, 35]. Systemic infection may trigger circulatory T cell and monocyte recruitment, which are the primary sources of IL‐17, IL‐6 and GM‐CSF, which can promote the migration of monocytes across the BBB [36]. Previous studies have shown that T‐cell recruitment is also impaired in AD in the presence of systemic infection [25].

Iba1+ and HLA‐DR+ contact VAM area correlated with a reduction in anti‐inflammatory cytokines IL‐10 and IL‐13 in AD. In addition, HLA‐DR+ VAM density also correlated inversely with reduced levels of IL‐6 [37, 38], which can exert anti‐inflammatory actions in a context‐dependent manner, and IL‐2 which has a protective role against AD pathology in APP mice [39]. These inverse relationships were also present and stronger in microglia in the proximity and parenchymal regions. In contrast, Iba1+ VAM correlated positively with proinflammatory cytokines, such as GM‐CSF, IL‐6 and IL‐23 in AD with systemic infection. In addition, HLA‐DR+ VAM also correlated with GM‐CSF and IL‐17, in AD with systemic infection. These relationships were strongest in the contact zone and became weaker further from the vessel. The findings indicate that VAM activation and endothelial injury are influenced by altered inflammatory homeostasis resulting in a net shift towards proinflammation; however, the cytokines and pathways involved may differ in AD and AD + SI. In contrast, CD68+ VAM area was elevated specifically in AD, was unchanged in the presence of systemic infection, and correlations with brain cytokine levels were sparse.

Many of the cytokines associated with increased density of Iba1+ and HLA‐DR+ VAM in AD in this study have previously been shown to be elevated in AD and implicated in the pathophysiology of cerebrovascular injury independently of Aβ levels [4]. IL‐6 [13] and IL‐17A [40, 41] are major drivers of cognitive decline and disease pathology in mouse models of AD and both stimulate microglial migration towards the cerebral vasculature. IL‐17 is elevated in AD and has previously been shown to induce BBB leakiness associated with reduced tight junction protein expression, and enhanced chemokine and adhesion molecule expression, associated with endothelial injury [42]. Injured microglia, via the release of pro‐inflammatory cytokines, also attract the infiltration of monocytes indicating a bi‐directional relationship between CNS and peripheral immune cells [43]. The infiltration of T‐cells, specifically Th17 cells, is a major source of IL‐17, a known activator of brain‐resident microglia, which contributes to localised neuroinflammation and BBB leakiness in Alzheimer's disease [44].

Few human studies have explored the distribution and activation status of VAM in relation to markers of vascular dysfunction in AD. In the present study, we observed that Iba1+ VAM correlated positively with brain fibrinogen level, a marker of BBB leakiness, in controls. This relationship was absent in the presence of systemic infection, and in AD with or without systemic infection. These data potentially suggest that microglia contribute to BBB breakdown at a very early disease stage and become dysregulated with AD and infection. We also observed that Iba1+ VAM correlated positively with VEGF‐A and endothelin‐1 expression in controls with SI. We previously reported that elevated VEGF‐A and endothelin‐1 were related to markers of reduced perfusion, elevated BBB leakiness, and Aβ_1‐42_, in the precuneus in the early stages of AD [45, 46]. Together, the observations suggest that Iba1+ VAM may contribute to pathogenic angiogenesis and vascular remodelling in early stage AD.

HLA‐DR+ VAM correlated inversely with MAP:PLP, a proxy marker of cerebral hypoperfusion, in controls and positively with MAG:PLP1 and VEGF‐A, a marker of acute cerebral ischemia, in AD. CD68+ VAM correlated positively with ICAM‐1 in Con + SI and inversely with E‐selectin in AD. Capillary stalling, associated with low‐level inflammation as a result of the upregulation of endothelial cell adhesion molecules, such as ICAM‐1, VCAM‐1 and E‐selectin, and entrapment of blood leucocytes, contributes to reduced blood flow in AD [47]. Although causality cannot be inferred, these data support the potential role of microglia contributing (or responding) to impaired cerebral blood flow under conditions of chronic inflammation.

The study has some limitations. The ImageJ plugin was used to assess VAM area rather than VAM number. It is likely that single microglial cells spanned our pre‐defined boundaries and contributed to signals within the contact and proximity zones. Perivascular macrophages (PVMs) play a central role in cerebrovascular dysfunction in neurodegenerative disease [48]. They share similar markers with VAM and can migrate towards and repair endothelial lesions [49]. In preclinical models, circulating monocytes penetrate the brain, via a damaged and leaky BBB, in conditions such as AD and can differentiate and replenish PVMs [48], or transform into monocyte‐derived microglia [50]. Microglia, perivascular macrophages and monocytes all share overlapping markers and morphology and contribute to BBB leakiness and AD pathogenesis [51]. Although we adopted a strict threshold to analyse microvessels less than 10 μm in size, in which PVMs do not normally reside, it will be important in future studies to precisely confirm the identity and phenotype of VAM using more sophisticated approaches such as spatial transcriptomics. It was not possible to assess the type and duration of systemic infection in relation to VAM distribution, which should be a focus of larger independent studies. This study also involved older adults in which VAMs may already be dysregulated, so comparisons in younger adults may also be informative. Lastly, the methodological approach in this study was focused on characterising changes in VAM labelling in proximity to vessels, and was not set up to assess changes in relation to plaque density within parenchyma, which is commonly reported to be elevated in AD in proximity to Aβ plaque accumulation [52, 53, 54].

## 5 CONCLUSION

In summary, our findings indicate that VAM are likely to contribute to cerebrovascular dysfunction in AD and may be an important independent contributor to cerebral blood flow dysregulation and BBB leakiness. Although observations from human post‐mortem tissue cannot infer causality, the relationships we report support a pathophysiological role of microglia in relation to cerebrovascular breakdown in AD that is influenced by systemic infection.

## AUTHOR CONTRIBUTIONS

OM, RF, and JSM devised the original study and experimental plan. OM generated, collected and analysed the data and prepared the data for publication. DJA measured brain cytokine levels and vascular markers. SC wrote the code for the ImageJ plug‐in. OM, RF, DB and JSM wrote and edited the manuscript and prepared it for publication.

## FUNDING INFORMATION

OM is supported by a University of Bristol PhD Scholarship award. RF is supported by an Alzheimer's Society Fellowship. J.S.M. is supported by an ARUK Senior Fellowship award (ARUK‐SRF‐2019A‐001).

## CONFLICT OF INTEREST STATEMENT

There are no conflicts of interest to declare.

## ETHICS STATEMENT

The study was approved by the Southwest Dementia Brain Bank (SWDBB) management committee (Human Tissue Authority licence number 12273) under the terms approved by the Bristol (18/SW/0029) ethical research committee.

## Supporting information


**FIGURE S1:** Representative images of CD31, CD68, and Iba1 staining across cohorts and brain regions. AD, Alzheimer's disease; AD + SI, Alzheimer's disease with the presence of systemic infection; CD31, cluster of differentiation 31; CD68, cluster of differentiation 68; Con, control; Con + SI, control with the presence of systemic infection; Iba1, ionised calcium‐binding adaptor molecule 1.
**FIGURE S2:** Representative images of CD31, HLA‐DR and Iba1 staining across cohorts and brain regions. AD, Alzheimer's disease; AD + SI, Alzheimer's disease with the presence of systemic infection; CD31, cluster of differentiation 31; Con, control; Con + SI, control with the presence of systemic infection; HLA‐DR, human leukocyte antigen‐DR; Iba1, ionised calcium‐binding adaptor molecule 1.
**FIGURE S3:** Vessel identification macro installation instructions. Full instructions on how to download the ImageJ macro utilised to identify vessels and quantify microglia proximity to the vessels.
**FIGURE S4:** CD68+ VAMs are increased in AD and in the presence of systemic infection. (a) Bar chart showing that the overall area of CD68+ density with pre‐defined regions in Con, Con+SI, AD, and AD+SI (*n* = 15 per group). (b) Bar chart showing CD68+ densities within the temporal cortex. (c) Bar chart showing CD68+ densities within the underlying white matter. AD, Alzheimer's disease; AD + SI, Alzheimer's disease with the presence of systemic infection; CD68+, cluster of differentiation 68 positive; Con, control; Con + SI, control with the presence of systemic infection; VAM, vessel‐associated microglia. *p < 0.05. **p < 0.01. ***p < 0.001. ****p < 0.0001.
**FIGURE S5:** Heatmaps showing the relationships between unadjusted CD68+ labelling, brain cytokine levels and biochemical markers of cerebrovascular function. Heatmaps illustrate the relationship between unadjusted CD68+ labelling of VAMs for (a) brain cytokine levels and (b) biochemical markers of cerebrovascular function in each pre‐defined regions. Aβ42, Amyloid beta 42; AD, Alzheimer's disease; AD + SI, Alzheimer's disease with the presence of systemic infection; Ang‐II, Angiotensin II; CD31, cluster of differentiation 31; CD68, cluster of differentiation 68; CLDN5, Claudin‐5; Con, control; Con + SI, control with the presence of systemic infection; EDN1, endothelin‐1; GM‐CSF, granulocyte‐macrophage colony‐stimulating factor; ICAM1, intracellular adhesion molecule‐1; IFNγ, interferon gamma; IL‐, interleukin; MAG:PLP, myelin‐associated glycoprotein:proteolipid protein; PDGFRβ, platelet‐derived growth factor receptor beta; TNFα, tumour necrosis factor alpha; VCAM‐1, vascular cell adhesion protein 1; VEGF‐A, vascular endothelial growth factor‐A. *p < 0.05. **p < 0.01.

**TABLE S1:** UK Brain Bank identification numbers. Table shows the unique UK Brain bank ID numbers to identify cases used in this study stratified into the four study groups. Abbreviations: AD, Alzheimer's disease; AD + SI, Alzheimer's disease with terminal systemic infection; Con, control; Con + SI, control with terminal systemic infection.
**TABLE S2:** Demographic and clinical features of cohorts. A table detailing the general demographic information and clinically diagnosed features of each cohort. Abbreviations: AD, Alzheimer's disease; AD + SI, Alzheimer's disease with terminal systemic infection; APOE −/−, indicates absence of ε4 allele and possession of either ε2 or ε3; Con, control; Con + SI, control with terminal systemic infection; F, female; M, male; n/a, not applicable; SD, standard deviation.

## Data Availability

Clinical and neuropathological data are linked to the UK Brain Bank Network (UKBBN) by a unique numeric UKBBN identifier.
