# Supplementary material for: Vessel‐associated microglia are differentially activated and distributed in relation to systemic infection and Alzheimer's disease
Source: Brain Pathol. 2025 Nov 30;36(3):e70052. doi: 10.1111/bpa.70052 (PMC13051992; doi:10.1111/bpa.70052)
Supplement: Supplementary file 1 — FIGURE S1: Representative images of CD31, CD68, and Iba1 staining across cohorts and brain regions. AD, Alzheimer's disease; AD + SI, Alzheimer's disease with the presence of systemic infection; CD31, cluster of differentiation 31; CD68, cluster of differentiation 68; Con, control; Con + SI, control with the presence of systemic infection; Iba1, ionised calcium‐binding adaptor molecule 1. FIGURE S2: Representative images of CD31, HLA‐DR and Iba1 staining across cohorts and brain regions. AD, Alzheimer's disease; AD + SI, Alzheimer's disease with the presence of systemic infection; CD31, cluster of differentiation 31; Con, control; Con + SI, control with the presence of systemic infection; HLA‐DR, human leukocyte antigen‐DR; Iba1, ionised calcium‐binding adaptor molecule 1. FIGURE S3: Vessel identification macro installation instructions. Full instructions on how to download the ImageJ macro utilised to identify vessels and quantify microglia proximity to the vessels. FIGURE S4: CD68+ VAMs are increased in AD and in the presence of systemic infection. (a) Bar chart showing that the overall area of CD68+ density with pre‐defined regions in Con, Con+SI, AD, and AD+SI (n = 15 per group). (b) Bar chart showing CD68+ densities within the temporal cortex. (c) Bar chart showing CD68+ densities within the underlying white matter. AD, Alzheimer's disease; AD + SI, Alzheimer's disease with the presence of systemic infection; CD68+, cluster of differentiation 68 positive; Con, control; Con + SI, control with the presence of systemic infection; VAM, vessel‐associated microglia. *p < 0.05. **p < 0.01. ***p < 0.001. ****p < 0.0001. FIGURE S5: Heatmaps showing the relationships between unadjusted CD68+ labelling, brain cytokine levels and biochemical markers of cerebrovascular function. Heatmaps illustrate the relationship between unadjusted CD68+ labelling of VAMs for (a) brain cytokine levels and (b) biochemical markers of cerebrovascular function in each pre‐defined regions. Aβ4 [file BPA-36-e70052-s002.docx]

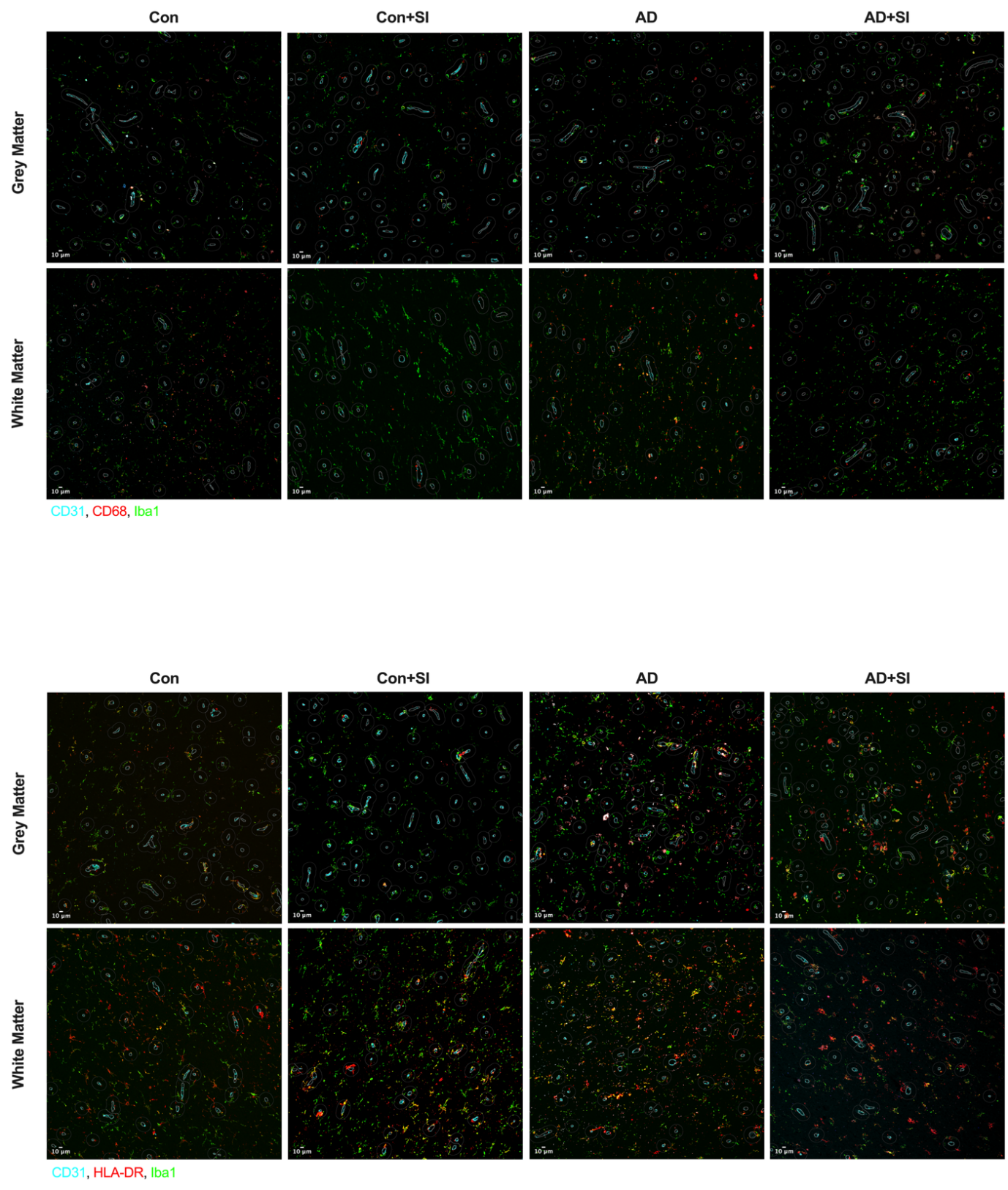


**Supplementary Figure 1. Representative images of CD31, CD68 and Iba1 staining across cohorts and brain regions.** Abbreviations: AD = Alzheimer’s disease. AD+SI = Alzheimer’s disease with the presence of systemic infection. CD31 = Cluster of differentiation 31. CD68 = Cluster of differentiation 68. Con = Control. Con+SI = Control with the presence of systemic infection. Iba1 = Ionised calcium-binding adaptor molecule 1.


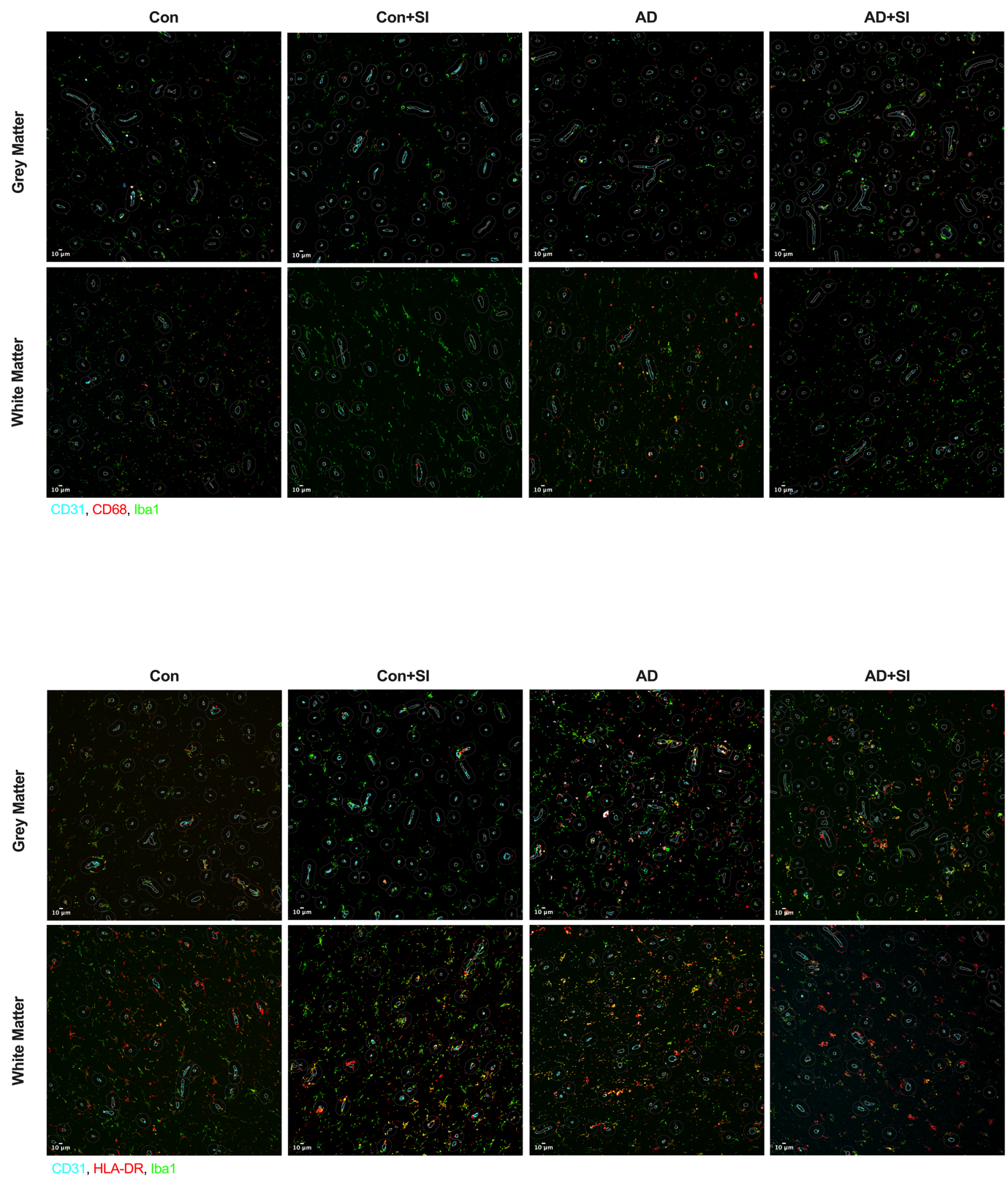


**Supplementary Figure 2. Representative images of CD31, HLA-DR and Iba1 staining across cohorts and brain regions.** Abbreviations: AD = Alzheimer’s disease. AD+SI = Alzheimer’s disease with the presence of systemic infection. CD31 = Cluster of differentiation 31. Con = Control. Con+SI = Control with the presence of systemic infection. HLA-DR = Human leukocyte antigen-DR. Iba1 = Ionised calcium-binding adaptor molecule 1.

The vessel analysis was conducted using the ModularImageAnalysis (MIA; https://mianalysis.github.io) plugin for ImageJ/Fiji. This is a plugin in which image and object analysis workflows can be assembled using a graphical environment and subsequently run on batches of images.

The specific workflow configured for this analysis is provided in the supporting information. This was configured using version 1.5.1 of the MIA plugin, but since future updates to ImageJ/Fiji and MIA may break compatibility with the workflow, it is best to prepare a copy of Fiji using the steps below as this emulates a known working configuration:

1. Download the relevant copy of Fiji (version dated 2024-02-01) from https://downloads.imagej.net/fiji/archive/20240201-1617/

2. Extract the Zipped archive to a location with read/write access (e.g. local "Documents" folder)

3. Download the core MIA plugin (version 1.5.1) from https://github.com/mianalysis/mia/releases/download/v1.5.1/MIA_-1.5.1.jar

4. Place the downloaded "MIA_-1.5.1.jar" file in the "plugins" directory of the downloaded Fiji (on Mac it is necessary to right-click the "Fiji" icon and select "Show package contents")

5. Download the MIA dependencies (version 1.5.1) from https://github.com/mianalysis/mia/releases/download/v1.5.1/mia-dependencies-1.5.1.zip

6. Extract the "mia-dependencies-1.5.1.zip" Zipped archive and place the "mia-dependencies" folder inside the "jars" directory of the downloaded Fiji

7. Download the MorphoLibJ plugin from https://github.com/ijpb/MorphoLibJ/releases/download/MorphoLibJ_-1.6.4/MorphoLibJ_-1.6.4.jar

8. Place the downloaded "MorphoLibJ_-1.6.4.jar" file in the "plugins" directory of the downloaded Fiji.

9. Download the DeepImageJ plugin from https://github.com/deepimagej/deepimagej-plugin/releases/download/2.1.16/DeepImageJ_-2.1.16.jar

10. Place the downloaded "DeepImageJ_-2.1.16.jar" file in the "plugins" directory of the downloaded Fiji.

11. Download the DeepImageJ dependencies from https://github.com/deepimagej/deepimagej-plugin/releases/download/2.1.16/dependencies_2116.zip

12. Extract the "dependencies_2116.zip" Zipped archive and place it in the "jars" directory of the downloaded Fiji such that the folder structure is /jars/dependencies_2.1.16/[files]

13. Create a folder called "models" in the top level of the downloaded Fiji (such that it's at the same level as folders such as "jars" and "plugins").

14. Download the "2024-04-24_Vessels_20x_RGB.zip" file from the supporting information of this paper

14. Extract the "2024-04-24_Vessels_20x_RGB.zip" Zipped archive and copy the "2024-04-24_Vessels_20x_RGB" folder to the "models" folder that was just created.

To run the workflow, start Fiji and go to "Plugins > ModularImageAnalysis (MIA) > MIA" then click "Load" when in the plugin and select the workflow file (.mia extension) included with the supporting information. A series of controls should appear allowing selection of the input file or folder. If selecting a folder, only files matching the "Extension" filter will be processed. To start the workflow running, click the "Run" button. A progress bar will move along the bottom of the plugin window and say "Complete!" when everything has finished.

For first runs on an image, "Detection mode" should be set to "Detect new", but for subsequent runs, this can be changed to "Load from file (.zip)", whereby the previously-detected objects will be loaded from automatically generated Zipped archives of ImageJ ROIs. When loading from file, it's also possible to enable a manual editing step in which the vessels can be edited using ImageJ's standard painting tools.

Files will be saved to the same location as the inputs.

**Supplementary Figure 3. Vessel identification macro installation instructions.** Full instructions on how to download the ImageJ macro utilised to identify vessels and quantify microglia proximity to the vessels.


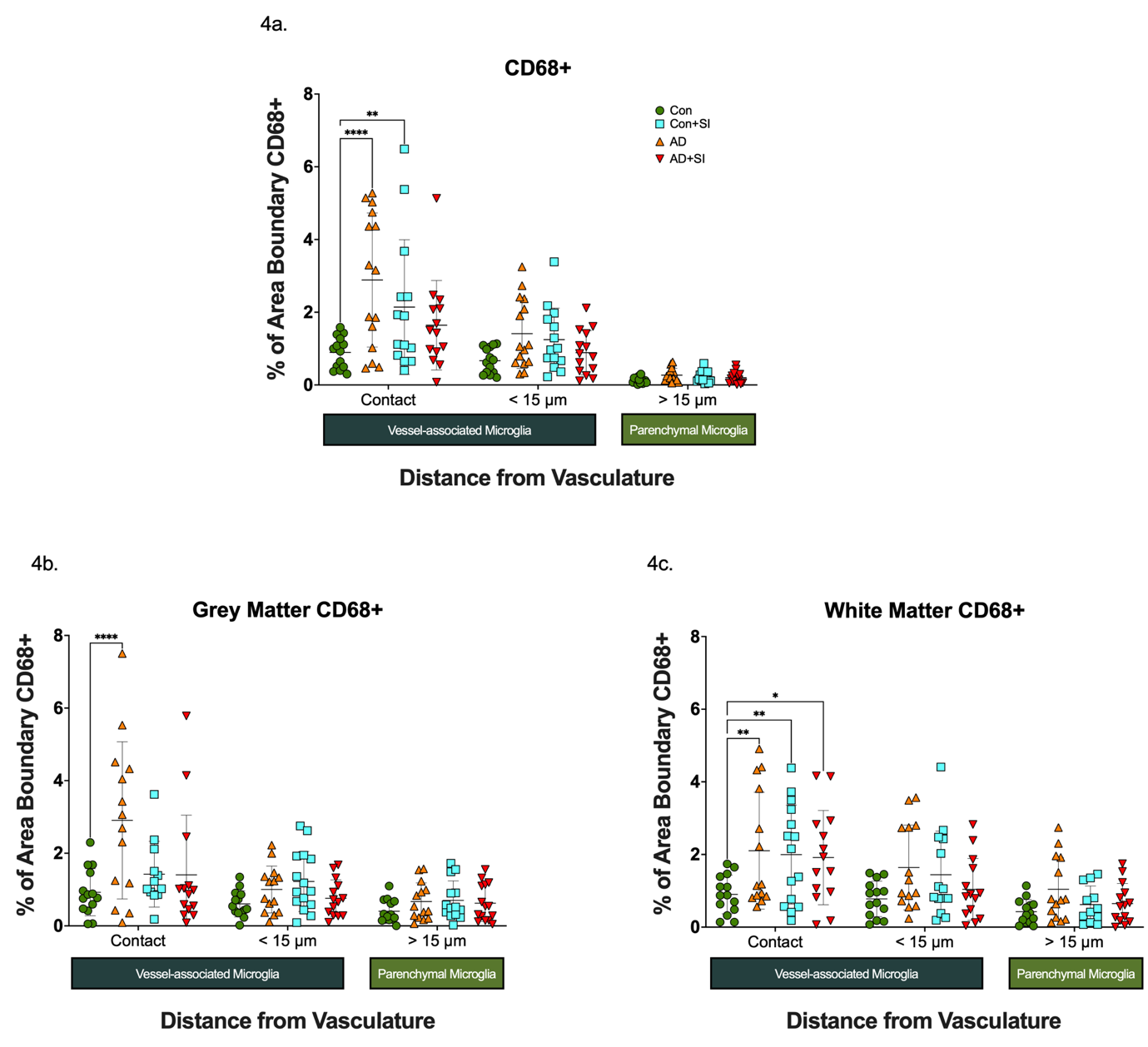


**Supplementary Figure 4:** **CD68+ VAMs are increased in AD and in the presence of systemic infection**. (**a**) Bar chart showing that the overall area of CD68+ density with pre-defined regions in Con, Con+SI, AD, and AD+SI (*n* = 15 per group). (**b**) Bar chart showing CD68+ densities within the temporal cortex. (**c**) Bar chart showing CD68+ densities within the underlying white matter. Abbreviations: AD = Alzheimer’s disease. AD+SI = Alzheimer’s disease with the presence of systemic infection. CD68+ = Cluster of differentiation 68 positive. Con = Control. Con+SI = Control with the presence of systemic infection. VAM = vessel-associated microglia. * = p < 0.05. ** = p < 0.01. *** = p < 0.001. **** = p < 0.0001


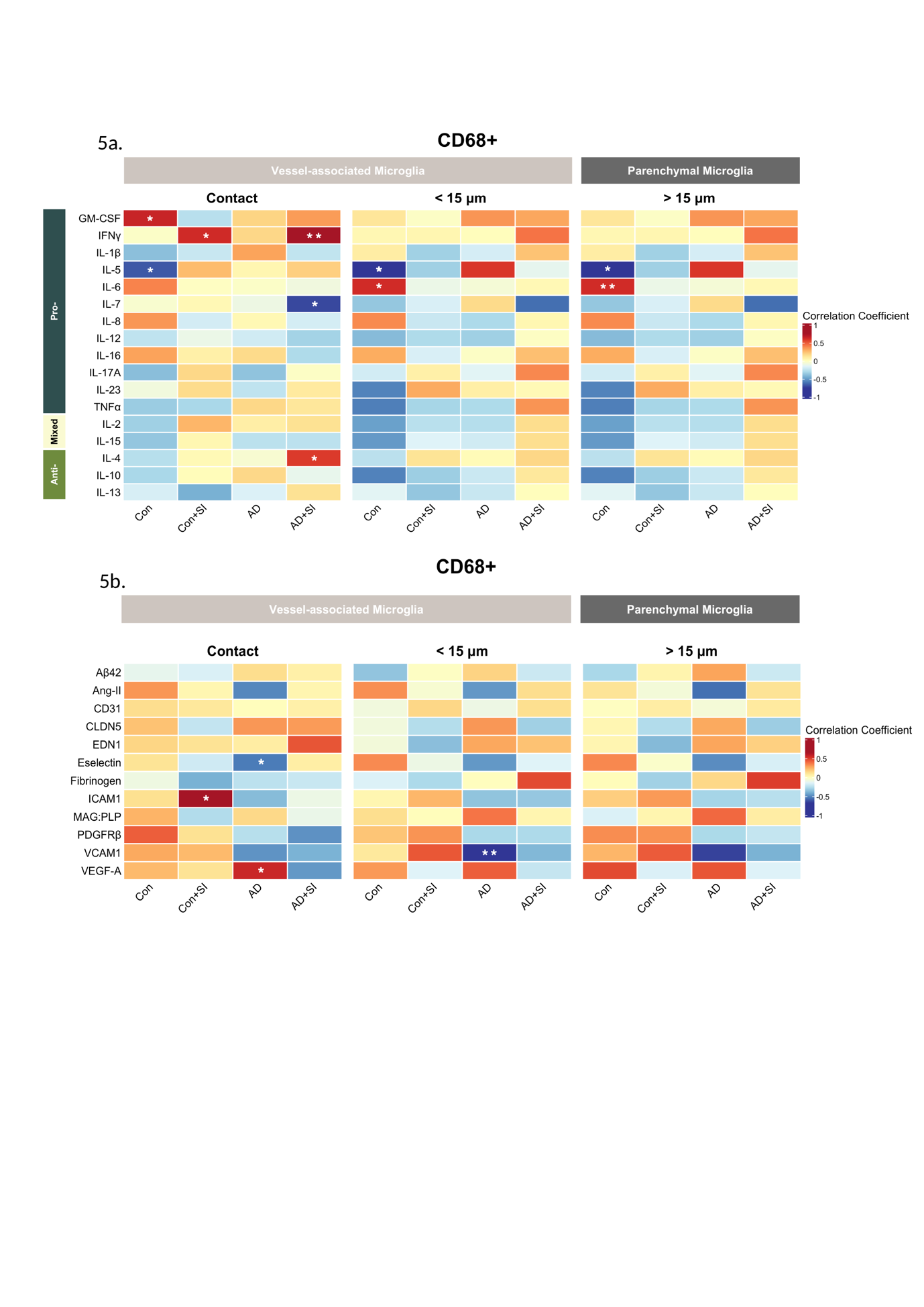


**Supplementary Figure 5: Heatmaps showing the relationships between unadjusted CD68+ labelling, brain cytokine levels and biochemical markers of cerebrovascular function**. Heatmaps illustrate the relationship between unadjusted CD68+ labelling of VAMs for **(a)** brian cytokine levels **(b)** biochemical markers of cerebrovascular function in each pre-defined regions. Abbreviations: Aβ42 = Amyloid beta 42. AD = Alzheimer’s disease. AD+SI = Alzheimer’s disease with the presence of systemic infection. Ang-II = Angiotensin II. CD31 = Cluster of differentiation 31. CD68 = Cluster of differentiation 68. CLDN5 = Claudin-5. Con = Control. Con+SI = Control with the presence of systemic infection. EDN1 = Endothelin-1. GM-CSF = Granulocyte-macrophage colony-stimulating factor. ICAM1 = Intracellular adhesion molecule-1. IFNγ = Interferon gamma. IL- = Interleukin. MAG:PLP = Myelin-associated glycoprotein:proteolipid protein. PDGFRβ = Platelet-derived growth factor receptor beta. TNFα = Tumour necrosis factor alpha. VCAM-1 = Vascular cell adhesion protein 1. VEGF-A = Vascular endothelial growth factor-A * = p < 0.05. ** = p < 0.01.
