# Supplementary material for: Vessel‐associated microglia are differentially activated and distributed in relation to systemic infection and Alzheimer's disease
Source: Brain Pathol. 2025 Nov 30;36(3):e70052. doi: 10.1111/bpa.70052 (PMC13051992; doi:10.1111/bpa.70052)
Supplement: Supplementary file 2 — TABLE S1: UK Brain Bank identification numbers. Table shows the unique UK Brain bank ID numbers to identify cases used in this study stratified into the four study groups. Abbreviations: AD, Alzheimer's disease; AD + SI, Alzheimer's disease with terminal systemic infection; Con, control; Con + SI, control with terminal systemic infection. TABLE S2: Demographic and clinical features of cohorts. A table detailing the general demographic information and clinically diagnosed features of each cohort. Abbreviations: AD, Alzheimer's disease; AD + SI, Alzheimer's disease with terminal systemic infection; APOE −/−, indicates absence of ε4 allele and possession of either ε2 or ε3; Con, control; Con + SI, control with terminal systemic infection; F, female; M, male; n/a, not applicable; SD, standard deviation. [file BPA-36-e70052-s001.docx]

| **Cases** | Con | Con+SI | AD | AD+SI |
| --- | --- | --- | --- | --- |
| **UK Brain Bank ID Number** | BBN_10251 | BBN_19608 | BBN_10252 | BBN_8917 |
|  | BBN_19613 | BBN_24325 | BBN_19615 | BBN_9037 |
|  | BBN_22623 | BBN_24337 | BBN_24330 | BBN_9076 |
|  | BBN_4205 | BBN_26009 | BBN_4215 | BBN_9123 |
|  | BBN_8964 | BBN_4229 | BBN_4216 | BBN_9150 |
|  | BBN_9292 | BBN_9028 | BBN_4232 | BBN_9242 |
|  | BBN_9340 | BBN_9329 | BBN_8915 | BBN_9295 |
|  | BBN_9344 | BBN_9359 | BBN_8968 | BBN_9317 |
|  | BBN_9354 | BBN_9392 | BBN_9118 | BBN_9326 |
|  | BBN_9365 | BBN_9408 | BBN_9336 | BBN_9341 |
|  | BBN_9407 | BBN_9422 | BBN_9361 | BBN_9343 |
|  | BBN006.29018 | BBN006.26096 | BBN_9367 | BBN_9346 |
|  | BBN006.30165 | BBN006.28893 | BBN_9371 | BBN_9372 |
|  | BBN006.30198 | BBN006.29470 | BBN_9395 | BBN_9379 |
|  | BBN006.32821 | BBN006.31516 | BBN_9426 | BBN_9433 |

**Supplementary Table 1. UK Brain Bank identification numbers.** Table shows the unique UK Brain bank ID numbers to identify cases used in this study stratified into the four study groups. Abbreviations: AD = Alzheimer’s disease. AD+SI = Alzheimer’s disease with terminal systemic infection. Con = Control. Con+SI = Control with terminal systemic infection.

| **Cases** | Con | Con+SI | AD | AD+SI |
| --- | --- | --- | --- | --- |
|  | (*n* = 15) | (*n* = 15) | (*n* = 15) | (*n* = 15) |
| **Gender** | 8F:7M | 7F:8M | 8F:7M | 9F:6M |
| **Age of death, years** (Mean±SD) | 86.2 ± 6.2 | 86.2 ± 7.4 | 83.5 ± 4.9 | 84.0 ± 5.3 |
| **Age of AD onset, years** (Mean±SD) | n/a | n/a | 74.3 ± 5.9 | 74.6 ± 8.5 |
| **Duration of AD, years** (Mean±SD) | n/a | n/a | 7.5 ± 2.8 | 7.6 ± 4.5 |
| **Post-mortem delay, hours** (Mean±SD) | 40.1 ± 20.7 | 46.7 ± 13.5 | 39.4 ± 20.3 | 40.8 ± 17.0 |
| **Braak stage, *n*** |  |  |  |  |
| 0-II | 15 | 15 |  |  |
| III-IV |  |  | 10 | 10 |
| V-VI |  |  | 5 | 5 |
| **Primary cause of death, *n*** |  |  |  |  |
| Cardiovascular | 6 | 1 | 5 |  |
| Cancer | 4 |  | 2 |  |
| Bronchopneumonia |  | 7 |  | 11 |
| Septicaemia |  | 2 |  | 2 |
| Other, non-infection | 5 | 2 | 8 |  |
| Other, infection |  | 3 |  | 2 |
| **APOE genotype, *n*** |  |  |  |  |
| APOE -/- | 11 | 12 | 6 | 6 |
| APOE ε4/- | 3 | 3 | 6 | 7 |
| APOE ε4/ε4 | 1 |  | 3 | 2 |

**Supplementary Table 2. Demographic and clinical features of cohorts.** A table detailing the general demographic information and clinically diagnosed features of each cohort. Abbreviations: AD = Alzheimer’s disease. AD+SI = Alzheimer’s disease with terminal systemic infection. APOE -/- = indicates absence of ε4 allele and possession of either ε2 or ε3. Con = Control. Con+SI = Control with terminal systemic infection. F = Female. M = Male. n/a = Not applicable. SD = Standard deviation.
